# The Regulatory Roles of RNA-Binding Proteins in Plant Salt Stress Response

**DOI:** 10.3390/plants14091402

**Published:** 2025-05-07

**Authors:** Tangying Wang, Kaiyuan Meng, Zilin Zhu, Linxuan Pan, Thomas W. Okita, Laining Zhang, Li Tian

**Affiliations:** 1 Collaborative Innovation Center for Efficient and Green Production of Agriculture in Mountainous Areas of Zhejiang Province, College of Horticulture Science, Zhejiang A&F University, Hangzhou 311300, China; 2022114012024@stu.zafu.edu.cn (T.W.); 949698@stu.zafu.edu.cn (K.M.); zhuzilin20250810@163.com (Z.Z.); 2024203011005@stu.zafu.edu.cn (L.P.); 2 Key Laboratory of Quality and Safety Control for Subtropical Fruit and Vegetable, Ministry of Agriculture and Rural Affairs, Zhejiang A&F University, Hangzhou 311300, China; 3 Institute of Biological Chemistry, Washington State University, Pullman, WA 99164, USA; okita@wsu.edu

**Keywords:** RNA-binding proteins, abiotic stress, salt stress

## Abstract

Salt stress is one of the most prominent abiotic stresses. Behind the intricate adaptive responses of plants to salt stress, the regulation of gene expression assumes a pivotal role. Complementing transcriptional mechanisms, post-transcriptional regulation performed by RNA-binding proteins provides an additional layer of control through sophisticated molecular machinery. RBPs interact with both RNA molecules and protein partners to coordinate RNA metabolism and, thus, fine-tune the expression of salt-responsive genes, enabling plants to rapidly adapt to ionic challenges. This review systematically evaluates the functional roles of RBPs localized in distinct subcellular compartments, including nuclear, cytoplasmic, chloroplastic, and mitochondrial systems, in mediating post-transcriptional regulatory networks under salinity challenges. Specific classes of RBPs are discussed in detail, including glycine-rich RNA-binding proteins (GR-RBPs), serine/arginine-rich splicing factors (SR proteins), zinc finger domain-containing proteins, DEAD-box RNA helicases (DBRHs), KH domain-containing proteins, Pumilio domain-containing proteins (PUMs), pentatricopeptide repeat proteins (PPRs), and RBPs involved in cytoplasmic RNA granule formation. By integrating their subcellular localization and current mechanistic insights, this review concludes by summarizing the current knowledge and highlighting potential future research directions, aiming to inspire further investigations into the complex network of RBPs in modulating plant responses to salt stress and facilitating the development of strategies to enhance plant salt tolerance.

## 1. Introduction

Soil salinity is one of the prominent forms of global land degradation. According to data reported by the Food and Agriculture Organization of the United Nations (FAO), the total area of salt-affected soils has reached nearly 1.4 billion hectares, accounting for 10.7% of the total global land area [1]. Natural factors such as arid climate, geological conditions, and the ascent of groundwater, along with human-induced factors including improper irrigation, excessive reclamation, and seawater intrusion, can all lead to soil salinization.

Salt stress is one of the most prominent abiotic stresses. It not only impacts biodiversity, accelerates land desertification, and gives rise to socioeconomic issues but also severely affects agricultural production through impeding plant development and slashing crop yields. At the seed germination stage, salt stress acts as an inhibitor [2,3]. It elevates osmotic pressure, thereby disrupting the water uptake essential for germination. Concurrently, it diminishes the levels of hormones such as gibberellin (GA) and indole-3-acetic acid (IAA), which are crucial for triggering germination. Moreover, salt stress retards the degradation of reserve substances and hampers protein/amino acid metabolism, all of which conspire to delay seed germination [4]. The high salt concentration in plants interferes with the absorption and metabolism of essential nutrients during plant growth stage. The excessive accumulation of Na^+^ and Cl^−^ disrupts the absorption of K^+^ and Ca^2+^ [5], which further cascades into disruptions in hormone synthesis and transport, ultimately exerting toxic effects on plant cells [6]. In addition, salt stress takes a toll on the photosynthetic apparatus of plants. It reduces stomatal conductance [7], depletes chlorophyll content [8], obstructs the photosynthetic electron transport chain, and leads to the over-accumulation of reactive oxygen species (ROS) [7,8]. These combined effects gradually impair the photosynthetic membrane system [9], culminating in a significant decline in plant photosynthesis. As a result, plants are unable to generate sufficient energy and materials to fuel their growth. Furthermore, salt stress can also disrupt normal respiration in plants. This disruption is characterized by a decreased respiration rate [10], mitochondrial damage [11], reduced activity of key respiratory enzymes, and altered accumulation of respiratory metabolites [12]. Collectively, the cumulative negative impacts of salt stress throughout the plant development cycle lead to symptoms such as slow and stunted growth, leaf yellowing, and, in severe cases, withering and death. Inevitably, these outcomes translate into a substantial reduction in crop yields. Given the gravity of these consequences, investigating the salt tolerance mechanisms of plants assumes paramount importance.

Plants have evolved multi-level adaptive strategies to cope with high-salt environments. These strategies span morphological/structural adaptations, physiological/biochemical regulations, hormone-mediated signaling, and gene expression modulation. Structurally, certain plant species exhibit distinct changes. Some plants augment root biomass and root–shoot ratios to enable them to more efficiently absorb water and nutrients from the saline soil [13], while some plants can modify their root architecture and growth direction in a phenomenon known as halotropism, which helps them avoid regions with high salt toxicity [14]. On the other hand, to curtail water loss under salt-induced osmotic stress, some plants reduce their leaf area, often developing succulent leaves [15], and also decrease stomatal conductance [16]. Physiologically, plants have developed several mechanisms to maintain cellular homeostasis. They accumulate compatible solutes such as proline and betaine to uphold cellular osmotic balance [17,18]. By adjusting the timing of xylem ion loading, plants can delay the transport of salts to the above-ground parts [19]. Ion homeostasis is regulated through transporters like high-affinity potassium transporters (HKTs), the plasma membrane salt overly sensitive (SOS) Na^+^/H^+^ exchanger, and the vacuolar membrane Na^+^/H^+^ exchanger (NHX), which are crucial for excluding sodium ions, sequestering them in vacuoles, and retaining potassium ions [20]. To scavenge ROS, plants activate both enzymatic and non-enzymatic antioxidant systems. Antioxidant enzymes such as superoxide dismutase (SOD), catalase (CAT), and ascorbate peroxidase (APX) are upregulated; meanwhile, reducing substances like ascorbic acid (AsA) and glutathione (GSH) are accumulated [20]. Hormone signaling pathways play a pivotal role in modulating these salt tolerance mechanisms. Under salt stress, the content of abscisic acid (ABA) in plants surges, which induces stomatal closure, reduces transpiration, and simultaneously triggers the expression of certain salt-tolerant genes, such as NHX1 and SOS3 [21]. The ethylene level also increases under salt stress. It is thought to enhance salt tolerance by activating stress-tolerant genes. For example, those encoding NAC transcription factors [7,21]. Auxin, cytokinin (CTK), and GA, are other hormones that come into play. Under salt stress, they indirectly enhance the plant’s salt tolerance capacity by regulating growth and development processes and photosynthesis efficiency [21,22]. Jasmonic acid (JA) and salicylic acid (SA) are involved in activating the plant defense system. In coordination with ABA, they jointly regulate the expression of antioxidant enzymes like SOD and CAT, which helps maintain redox balance within the plant cells [23].

Behind the intricate adaptive responses of plants to salt stress, the regulation of gene expression assumes a pivotal role. This regulatory process operates primarily at transcriptional and post-transcriptional levels. Transcriptional regulation is orchestrated by primarily transcription factors (TFs), which act as molecular switches controlling gene expression. For instance, basic helix–loop–helix (bHLH) TFs recognize and bind to the cis-acting elements within the promoter region of target genes, thereby activating downstream genes involved in critical physiological processes such as osmotic adjustment and ion homeostasis [24]. These regulatory proteins often engage in combinatorial interactions or integrate hormonal signaling pathways, collectively constructing elaborate transcriptional networks. Complementing these transcriptional mechanisms, post-transcriptional regulation provides an additional layer of control through sophisticated molecular machinery. It involves dynamic processes including RNA splicing, RNA modification, RNA stability, RNA transport and localization, and translational regulation [25,26,27]. Central to these processes are RNA-binding proteins (RBPs), which interact with both RNA molecules and protein partners to coordinate RNA metabolism. By governing these post-transcriptional events, RBPs fine-tune the expression of salt-responsive genes, enabling plants to rapidly adapt to ionic challenges [28,29]. Elucidating the mechanistic details of RBP-mediated post-transcriptional regulation under salinity stress holds profound implications for improving crop resilience and agricultural productivity.

Over the past two decades, a number of studies have identified RNA-binding proteins (RBPs) as critical regulators of plant adaptation to salt stress. This review systematically evaluates the functional roles of RBPs localized in distinct subcellular compartments, including nuclear, cytoplasmic, chloroplastic, and mitochondrial systems, in mediating post-transcriptional regulatory networks under salinity challenges. Particular emphasis is placed on summarizing recent advancements in the evolutionarily conserved protein families, such as glycine-rich RNA-binding proteins (GR-RBPs), serine/arginine-rich splicing factors (SR proteins), CCCH-type zinc finger proteins (TZF family), DEAD-box RNA helicases (DBRHs), KH domain-containing proteins, Pumilio domain-containing proteins (PUMs), and pentatricopeptide repeat proteins (PPRs). By integrating current mechanistic insights, this review highlights the emerging roles of RBPs as central hubs connecting environmental cues to transcriptome plasticity and proposes future research directions to address critical gaps in our understanding of post-transcriptional regulation under abiotic stress.

## 2. Classification of Plant RBPs

Plant RNA-binding proteins (RBPs) represent a structurally diverse and functionally versatile class of regulatory molecules ubiquitously present across plant kingdoms. These proteins mediate RNA interactions through evolutionarily conserved RNA-binding domains (RBDs) [30], forming dynamic ribonucleoprotein (RNP) complexes [31] that orchestrate RNA metabolism. The spatiotemporally regulated interactions between RBPs and single-/double-stranded RNAs are critical for RNA biogenesis [32], subcellular trafficking, and translational control processes essential for maintaining cellular homeostasis and enabling adaptive responses to developmental cues and environmental perturbations [33].

Advances in high-throughput methodologies, including mRNA interactome capture [34,35], large-scale proteomics, and integrative bioinformatics pipelines [33,36], have significantly accelerated RBP discovery. This has led to the identification of over 20 distinct RBD families in plants, which are categorized into several structurally defined classes, including RNA recognition motif (RRM), K homology (KH) domain, zinc finger domain (CCCH type), cold shock domain (CSD), double-stranded RNA-binding domain (DS-RBD), DEAD/DEAH RNA helicases, PUM RNA-binding domain, PPR, YT512-B homology (YTH) domain, S1 RNA-binding domain, chloroplast ribosome maturation and splicing domain (CRM), Piwi/Argonaute/Zwiklle (PAZ) domain, nuclear transport factor 2 domain, and like-Smith (commonly known as LSm) poly(A)-binding domains [37,38,39,40,41,42,43,44]. Building upon the preceding discussion, proteins containing canonical RBDs can be further subclassified based on their N- or C-terminal auxiliary motifs. Some of these motifs are rich in specific amino acids, accordingly named glycine-rich region, arginine-rich domain, arginine/glycine or arginine/glycine/glycine (RG/RGG) repeats, arginine/aspartic acid (RD) repeats, and serine/arginine (SR) or arginine/serine (RS)-rich domain [45]. While RBDs provide sequence-specific RNA recognition, auxiliary motifs mainly modulate protein stability, subcellular localization, and protein interactome dynamics. This modular architecture allows RBPs recognize and bind the specific mRNA untranslated regions (UTRs) or cis-acting elements and dictate their functional roles in processes ranging from pre-mRNA splicing to translational control.

**Figure 1:**
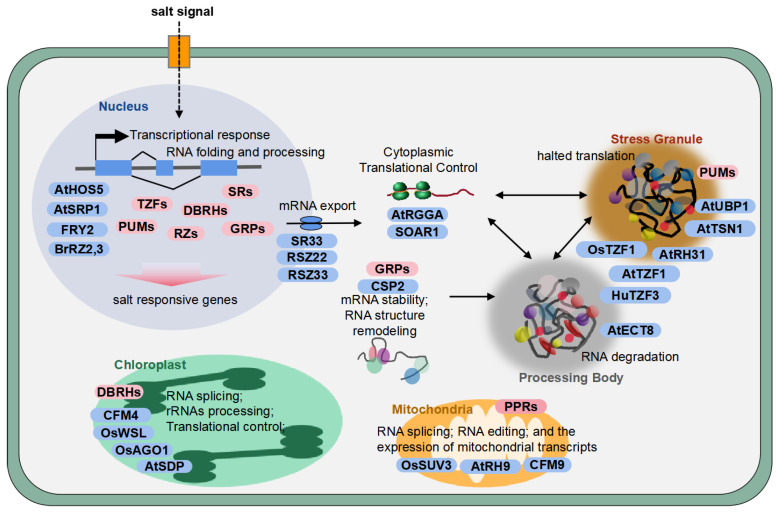
Model depicting the subcellular localization of typical RBPs involved in plant adaptation to salt stress. Salt stress may either activate or suppress the expression of RBPs. In the nucleus, RBPs can regulate RNA folding and RNA processing events and, thereby, modulate the expression of salt-responsive genes. Some specific RBPs may be involved in the nuclear export of mRNAs through regulating TREX (transcription–export) complex assembly. Once the mRNAs are exported into the cytoplasm, salt-responsive RBPs can regulate various processes. These include the translation of mRNAs, RNA degradation via processing bodies, and the storage of mRNAs in stress granules. Additionally, some RBPs can assist in RNA structure remodeling and contribute to the regulation of mRNA stability in cytoplasm. Certain RBPs are targeted to chloroplasts or mitochondria. In these organelles, they play a role in regulating RNA splicing, ribosomal RNA (rRNA) processing, and translational processes that are crucial for organelle biogenesis and plant adaptation to salt stress. More detailed information is presented in Table 1 and discussed in the main text. RBP families within the light-red box indicate that more than one RBP in the family has been identified in the cellular process. RBPs in the blue box represent a single RBP from a particular family.

Despite their structural diversity, RBPs exhibit highly specific subcellular localizations that underlie their functional roles in plant cells. The spatiotemporal regulation of RNA metabolism within distinct subcellular compartments, including nuclei, cytoplasm, chloroplasts, and mitochondria, is fundamentally associated with plant growth and adaptive responses. Deciphering how these spatially segregated RBPs coordinate post-transcriptional processes under salt stress is critical for understanding the molecular basis of plant salt adaptation. In the following sections, we will first systematically assess the functional roles of specific types of RBPs. These include GR-RBPs, SR/RS proteins, ZF domain-containing proteins, DBRHs, KH domain-containing proteins, and PUM proteins, especially those localize in nucleus and cytoplasm. Subsequently, we will specifically elucidate the RBPs involved in cytoplasmic RNA granule formation, as well as those participating in chloroplast and mitochondrial functions. The information regarding these RBPs is presented in Table 1 and Figure 1, which integrates their subcellular localizations with mechanistic insights, thereby highlighting their contributions to stress-responsive regulatory networks.

## 3. Functions of Salt-Responsive RBPs in the Nucleus and Cytoplasm

### 3.1. GR-RBPs

Among the characterized plant RBPs, glycine-rich RNA-binding proteins (GR-RBPs) represent a well-characterized family participating in plant defense systems induced by abiotic and biotic stress [62,113,114,115]. Belonging to the type IV glycine-rich protein (GRP) superfamily [113], GR-RBPs are characterized by the presence of an extra RNA recognition motif (RRM) or cold shock domain (CSD) motif at the N-terminus and a glycine-rich region at the C-terminus [116]. Based on domain composition, GR-RBPs are further subclassified into four evolutionary clades [62]. GR-RBPs in subgroup IVb contain an RRM domain and a CCHC zinc finger domain, and subgroup IVc possess a CSD domain and multiple CCHC zinc finger domains, while subgroup IVa and IVd contain a single- and two-tandem RRM domain, respectively [114]. GR-RBPs are reported to shuttle dynamically between the nucleus and cytoplasm, where they interact with target transcripts to modulate post-transcriptional processes [113,117].

The *Arabidopsis* genome harbors eight members of the GR-RBP family. When subjected to salt stress conditions, among these members, only *GRP1* experiences a significant upregulation within 24 h, while high salinity leads to the downregulation of *GRP3*, *GRP4*, *GRP6*, and *GRP7* [49]. Further analysis reveals that the overexpression of *GRP4* exhibits a detrimental effect on seed germination and seedling growth under salt stress [49]. By contrast, the heterogenous overexpression of *GRP1* orthologs from other species enhances *Arabidopsis* tolerance to salt stress. For example, seeds from the transgenic lines expressing the *Malus prunifolia MpGR-RBP1* germinated earlier under salt stress compared to those of the wildtype plants [50].

AtGRP7 belongs to subgroup IVa of GR-RBPs. Its overexpression alters the splicing landscape of 59 alternative splicing events [47,118]. Mechanistically, AtGRP7 binds to its own and other genes’ pre-mRNAs (e.g., *AtGRP8*), promoting the utilization of 5′ cryptic splice sites and generating unstable splicing variants that undergo nonsense-mediated mRNA decay (NMD). This autoregulatory feedback loop fine-tunes *AtGRP7* expression levels to maintain cellular homeostasis during salt stress [47,118]. Genetic evidence further supports this model, in which the *atgrp7-1* null mutant exhibits complete germination arrest under salt stress, highlighting AtGRP7 as a critical determinant of early seedling establishment in saline environments [43]. Intense alternative splicing of *AtGRP7* itself produces functionally distinct protein isoforms, including truncated or modified polypeptides, which may diversify stress responses through differential subcellular localization or RNA-binding specificity. Phylogenetic analysis reveals that Class IVa Branch I GR-RBPs, including AtGRP7, are evolutionarily conserved in rice, tomato, and sweet potato, with conserved alternative splicing patterns under salt stress [46]. Notably, orthologs of AtGRP7 exhibit species-specific regulatory roles. For example, *CsGRP7* from camelina (*Camelina sativa*) displays salt-induced upregulation, contrasting with the downregulation of *AtGRP7* in *Arabidopsis*. Heterologous expression of CsGRP7 in *Arabidopsis* increases salt sensitivity, suggesting divergent functional evolution between species. This discrepancy may arise from differences in cisregulatory elements or protein–protein interactions, highlighting the complexity of GR-RBP-mediated stress responses across plant taxa [54].

Another GR-RBP reported in *Arabidopsis* is AtGRDP2. The protein contains a potential RRM and a DUF1399 domain and is complemented by a short glycine-rich region. Although *AtGRDP2* transcripts are found to be primarily expressed in floral organs, its overexpression enhances *Arabidopsis* salt tolerance. This is manifested by a higher survival rate during the recovery stage after salt stress and induction of salt-responsive marker genes, such as *Responsive to Desiccation 29B* (*RD29B*), *Arabidopsis Early Methionine-Labeled 6* (*EM6/LEA*), and *ABRE Binding Factor 4* (*ABF4*). Conversely, in the mutant *Atgrdp2* lines, an obvious salt-stress-sensitive phenotype was observed [119]. Furthermore, heterologous expression of *AtGRDP2* in lettuce results in an abnormal ratio of chlorophyll a/b and improve lettuce salt tolerance. Under 150 mM NaCl, the transgenic lettuce plants display a phenotype of longer roots and increased fresh biomass [119]. Although the target RNAs of AtGRDP2 were not investigated in the study, it has been proposed that the function of AtGRDP2 in salt stress is linked to ABA due to the hypersensitivity to ABA exhibited by its overexpression lines.

Beyond *Arabidopsis*, GR-RBPs have also been studied in various other species. *BpGRP1* is significantly upregulated in stems and leaves after salt stress in birch (*Betula platyphylla*). The plants overexpressing *BpGRP1* exhibit higher growth rates, greener leaves, and less wilting under salt stress compared to WT plants [53]. Another study revealed that overexpression of *LbGRP1* in *Limonium bicolor* significantly alters the activities of CAT and SOD, the proline content, and the Na^+^/K^+^ ratio. This suggests that LbGRP1 might directly or indirectly regulate these physiological pathways through post-transcriptional regulation mechanisms under salt stress conditions [52]. Under salt treatment, the transcriptional level of *SbGR-RNP* from *Sorghum bicolor* increases by 4 to 7 times [56]. In *Malus hupehensis*, the transcriptional level of *MhGR-RBP1* in the leaves doubles after being treated with salt for 2 days compared to the initial level [55]. A glycine-rich RNA-binding protein, ZjGRP, induced by salt in *Zoysia japonica*, negatively regulates the seed germination and seedling growth of *Arabidopsis* under salt stress. Moreover, subcellular localization analysis indicates that ZjGRP is predominantly distributed between the nucleus and the cytoplasm of tobacco cells. Under salt treatment, ZjGRP is also capable of regulating the expression of certain genes related to salt stress [57]. A recent study has revealed that PeGRP2 in *Populus euphratica* is also involved in mRNA regulation under salt stress conditions. Its expression in the roots is found to be enhanced once salt exposure commences. RNA affinity purification (RAP) sequencing analysis reveals PeGRP2 interacts with mRNAs encoding photosynthetic proteins, antioxidative enzymes, ATPases, and Na^+^/H^+^ antiporters [58]. The transcript levels of these target RNAs dramatically decreased in the overexpression line of PeGRP2 upon application of NaCl [58], suggesting a negative regulatory role of PeGRP2 in salt stress response.

Due to the presence of RRM and zinc finger domains, GR-RBPs in subgroup IVb are also termed RZ proteins [61]. Multiple studies have reported that some RZ proteins are regulated by the ABA signaling pathway [60,120], suggesting a possible role of RZ proteins in plant stress response. Under salt stress conditions, AtRZ-1a functions as a negative regulator in the germination and subsequent growth of *Arabidopsis* [60]. Proteomic analysis of *AtRZ-1a* overexpression lines reveals that AtRZ-1a modulates the expression of several germination-responsive genes and proteins involved in maintaining the homeostasis and function of ROS compared to wildtype [60]. Besides the *Arabidopsis* RZ proteins, three members of the RZ family in the wheat genome also respond to salt stress. Under salt stress, seed germination in all three transgenic *Arabidopsis* lines overexpressing *TaRZ* was delayed compared to wildtype [62]. Among them, TaRZ2 and TaRZ3 play negative regulatory roles in seed germination and seedling growth of *Arabidopsis* under salt stress conditions, while TaRZ3 inhibits the seedling growth of *Arabidopsis* in the presence of ABA by regulating the transcriptional levels of germination-relevant genes and genes associated with the ABA signaling pathway [62]. It was proposed that RZs might act as RNA molecular chaperones [121]. They prevent RNA misfolding or resolve misfolded RNA structures, thereby promoting the RNA folding process, participating in RNA metabolism, and ultimately influencing the transcriptional levels of stress-responsive genes [113].

Plant cold shock domain proteins (CSDPs) represent a distinct subgroup IVc lineage, distinguished from bacterial cold shock proteins (CSPs) by their additional CCHC zinc finger motifs [63,122]. The CSD mediates dual nucleic acid binding to single-stranded RNA and DNA, regulating stress-responsive gene expression [44]. For instance, *Arabidopsis* AtCSP2 stabilizes stress-responsive mRNAs by binding their 3′UTRs under low temperatures [63]. Under salt stress, *Arabidopsis* CSDP2 enhances salt tolerance during seedling establishment [58]. However, the functions of CSDPs in plants have not been fully elucidated and their functions beyond temperature and salinity stresses are largely unexplored.

The regulation of mRNA stability represents the second crucial aspect where RBPs are involved in post-transcriptional regulation and concurrently respond to salt stress. RBPs bind to specific sequences, such as AU-rich elements (AREs) situated in the 3′ UTR of mRNA, forming a complex. This complex either recruits nucleases or impedes nucleases from degrading mRNA, thereby influencing the half-life of mRNA. For instance, AtGRP7 and AtGRDP2 enhance salt tolerance through regulating the expression of antioxidant enzymes like SOD and CAT. The regulation is achieved by their binding to the 3′UTR or coding region of these antioxidant enzyme genes, which can potentially regulate the stability of the corresponding mRNAs. As a result, these RBPs modulate the expression of these genes and ultimately reduces the accumulation of ROS [48].

There exist other GR-RBPs that localize to the cytoplasm and function as RNA chaperones, responding specifically to salt stress. For instance, the cytoplasmically localized SaRBP1 *(Suaeda asparagoides* RNA-binding protein 1) belongs to a distinct subclass of glycine-rich proteins (GRPs) in harboring three RGG boxes [123]. In vitro experiments demonstrated that GST-SaRBP1 binds to synthesized RNA in a concentration-dependent manner. Similar to *GRP4*, the overexpression of *SaRBP1* in *Arabidopsis* led to enhanced growth compared to wildtype seedlings under NaCl stress, although this effect was not observed during the germination stage [59]. However, the precise underlying mechanisms remain elusive.

### 3.2. SR/RS Proteins

High salinity stress is reported to promote the occurrence of alternative splicing of stress-responsive genes and affect the expression of the genes coding spliceosome components in many plant species [124,125,126,127,128,129,130,131,132]. Alternative splicing (AS) is orchestrated by the spliceosome, a dynamic macromolecular complex composed of five small nuclear ribonucleoproteins (snRNPs), namely, U1, U2, U4/U6, and U5, along with hundreds of accessory proteins. These accessory proteins include serine/arginine-rich-domain-containing proteins (SRs) and heterogeneous nuclear ribonucleoproteins (hnRNPs) [133,134]. SR proteins are crucial regulators of eukaryotic gene expression, governing both constitutive and alternative splicing processes within the nucleus [135,136]. Structurally, these proteins are defined by their modular organization. They feature one or multiple conserved N-terminal RRMs for RNA binding, and a variable C-terminal SR or RS domain that mediates protein–protein interactions [65,136,137]. Through these domains, SR proteins facilitate spliceosome recruitment to pre-mRNA splice sites, ensuring precise and efficient splicing. Plant SR proteins show evolutionary diversification. Based on domain architecture and sequence motifs, six distinct subfamilies have been identified. The six subfamilies include SR, RS, SC, SC-like (SCL), RSZ, and ES2Z [40,138,139]. Both SR and RS proteins are typically characterized by two canonical RRMs. However, the second RRM within RS proteins usually lack an SWQDLKD motif that contributes to a surface-mediated protein–protein interaction. Members of the SC and SCL subfamilies contain only one RRM. Notably, the SCL, together with SC35 (ortholog of human splicing factor SC35)-like proteins, is distinguished by a short charged N-terminal extension. RSZ and RS2Z proteins are named for the presence of arginine/serine-rich domain and one (RSZ) or two (RS2Z) CCHC zinc knuckle domains. Among them, three subfamilies, RS, SCL, and RS2Z, are plant-specific, emphasizing their unique contributions to plant splicing regulation [139]. A recent addition to this classification is the SR45 subfamily, which contains two RRMs and an RS domain but exhibits functional divergence from canonical SR proteins, suggesting specialized roles in plant stress responses [138].

Under salt stress, some SR/RS proteins themselves undergo alternative splicing, which changes their function and leads to the alteration of salt stress tolerance. For instance, in *Arabidopsis*, the SR-like protein AtSR45 undergoes stress-responsive AS to generate functionally distinct isoforms [65]. Loss-of-function *atsr45* mutants exhibit salt hypersensitivity throughout the lifecycle, particularly during seed germination [65]. The closely related AtSR45a protein contains two RS domains, and its AS produces two variants: *AtSR45a-1a* with deletion of C-terminus and *AtSR45a-1b* with full-length sequence [140]. Under salt stress, AtSR45a-1a interacts with the cap-binding complex (CBC) subunit CBP20 to modulate AS of salt-responsive genes, while AtSR45a-1b enhances the binding affinity of CBC [64]. Paradoxically, the loss of AtSR45a function confers salt tolerance during germination, while overexpression of either isoform increases sensitivity with dosage-dependent regulation [64]. In addition, AtSR45 may also participate in the regulation of mRNA stability by binding to the target RNA [141]. These findings highlight the intricate and context-dependent nature of SR/RS protein-mediated alternative splicing in modulating plant salt stress tolerance, where the balance between different isoforms and their interactions with other regulatory components plays a crucial role in determining the plant’s adaptive response to saline conditions.

Several studies have reported that certain SR/RS proteins play a crucial role in regulating plant tolerance to salt stress by modulating the splicing of stress-responsive genes. In rice, OsRS33 responds to external salt stress by splicing a set of pre-mRNAs, leading to nearly 600 differential splicing events [68]. In the context of heterologous expression, introducing *MeSR34* and *MeSR40* from Cassava (*Manihot esculenta*) into *Arabidopsis* has been demonstrated to improve the homeostasis of ROS and enhance the expression of genes related to osmotic adjustment [65,67]. Under salt stress conditions, MeRS40 can accurately recognize splicing sites. It does so either by influencing the turnover and transportation of specific splicing variants or by reducing the stability of the spliceosome complex [67]. However, the overexpression of *MeSR40* in its native host, cassava, inhibits the expression of its endogenous counterpart. This is due to the negative autoregulation of its own precursor mRNA, which in turn reduces the plant’s salt tolerance.

Pentatricopeptide repeat proteins (PPRs) are intricately associated with chloroplasts and mitochondria, a topic that will be elaborated on subsequently. The pre-mRNAs of certain *PPR* genes require alternative splicing (AS) in the nucleus to generate functional transcripts. In tomato, PPRs were found to be among the prominent genes undergoing AS during salt stress [132]. It is reported that OsRS33 may form a complex with other SR proteins, such as RS29, to collaboratively regulate the splicing efficiency of PPR transcripts [68,142].

AtSCL30a, a member of the plant-specific SCL subfamily, serves as a negative regulator of ABA signaling. It can promote seed germination under salt stress [69]. Similarly, heterologous expression of the *Manihot esculenta MeSCL30a* gene and *Populus trichocarpa PtSCL30* in *Arabidopsis* induces a hypersensitive response to salt during seed germination and seedling growth stages, respectively [70,71]. Conversely, GhSCL-8 in cotton can interact with GhSR from other subfamilies and plays a positive role under salt stress [72]. Collectively, while many specific regulatory mechanisms remain elusive, it is evident that SR proteins from diverse subfamilies synergistically regulate post-transcriptional gene regulation in response to salt stress. Specifically, they govern the splicing and alternative splicing of pre-mRNA, thereby influencing gene expression and protein function.

Beyond their fundamental function in RNA splicing, certain SR/RS proteins are also involved in mRNA transport process. After pre-mRNA splicing and processing, the resultant mature RNA is transported from the nucleus to the cytoplasm via the nuclear pore complex (NPC) [143]. To guarantee the accurate entry of mRNA into the cytoplasm for translation, various RBPs, which assemble into diverse mRNP complexes, play specialized roles in this process. Some SR proteins contribute to the assembly of mRNP complex, facilitating their docking with the NPC and enabling nucleocytoplasmic transport. In *Arabidopsis*, RSZ22 and RSZ33 have been found to co-localize with TEX1, a subunit of the transcription–export (TREX) complex [144]. The TREX complex plays a crucial role in coupling RNA synthesis and processing to the export of nuclear mRNA, ensuring the efficient transfer of mature transcripts out of the nucleus. Additionally, another protein, SR33, has been reported to co-localize with hyper recombination 1 (HPR1), yet another subunit of the TREX complex, within the nucleus of *Arabidopsis* cells [66]. These co-localization findings suggest that SR proteins and the TREX complex likely engage in physical interactions to jointly regulate mRNA export. The previously mentioned OsRS33, during salt stress, has a splicing function that is reported to rely on the mRNA export capacity of the TREX complex [68]. This indicates a potential link between the regulation of gene splicing under stress conditions and the efficiency of mRNA transport out of the nucleus. Furthermore, CONSTITUTIVE EXPRESSER OF PATHOGENESIS-RELATED GENES 5 (CPR5), an RBP belonging to the SR family, possesses an N-terminal nuclear localization signal (NLS) and is predominantly localized within the NPC [145]. CPR5 regulates the nucleocytoplasmic transport of mRNA in *Arabidopsis* [145]. Given its role in mRNA transport, it is reasonable to assume that CPR5 also participates in the plant’s response to salt stress through this transport pathway, potentially influencing the expression of stress-responsive genes by controlling the availability of their corresponding mRNAs in the cytoplasm.

### 3.3. Zinc Finger Domain-Containing RBPs

Zinc finger proteins can be classified into nine categories based on the number and arrangement of cysteine (Cys) and histidine (His) residues, namely, C2H2, C2HC, C2HC5, CCCH, C3HC4, C4, C4HC3, C6, and C8 [146]. In the plant kingdom, C2HC-type zinc finger proteins can collaborate with other RBDs to form proteins belonging to the RZ, CSDP, and SR families, while proteins of the TZF family comprise a subclass of CCCH-type zinc finger proteins. The core feature of TZF is the presence of two tandemly arranged C-X7-8-C-X5-C-X3 zinc finger domains, which are supplemented with a plant-specific arginine-rich (RR) motif located upstream of the TZF domain [147]. The RR motif is reported to be intimately associated with the RNA-binding affinity of TZF proteins [147,148]. Plant TZF proteins can be further categorized into two distinct groups, namely, the RR-TZF group that contains the arginine-rich (RR) and TZF domains and the ANK-RR-TZFs group that encompasses additional ankyrin repeat sequence (ANK) along with the RR-TZF domains. Among these, the ANK repeat motif is recognized as a protein–protein interaction motif and is pivotal in regulating plant growth and development processes [149].

Representative examples of TZF family proteins include the *Arabidopsis* RR-TZF family members AtTZF2 and AtTZF3, and salt-induced zinc fingers AtSZF1 and AtSZF2. While the expression of nucleus-localized AtTZF2 and AtTZF3 are induced by ABA treatment and various stresses including salt, their RNAi lines exhibited a slightly hypersensitive phenotype to salt stress [80]. Intriguingly, both proteins exhibit in vitro RNase activity [80], which implies their potential involvement in RNA metabolic processes. Although the expression of a number of genes involved in JA, ABA, and biotic/abiotic stress responses was altered in these RNAi lines, critical questions such as whether the nuclease activity is sequence-specific, whether this activity occurs in vivo, what their target RNAs are, and how they modulate the target RNA metabolism, remain unanswered. Under salt stress conditions, the *Arabidopsis atszf1-1/atszf2-1* double mutant exhibits a more sensitive response to salt stress in terms of seed germination and growth inhibition compared to *atszf1-1* and *atszf2-1* single mutants [76]. Curiously, the expression of a group of salt stress-responsive genes is increased in these single or double mutants but downregulated in the *AtSZF1* overexpression lines, suggesting a negative regulation by AtSZF1 and AtSZF2 on these genes. Further study is required to confirm the regulatory role of AtSZF1 and AtSZF2 in salt stress response.

Other proteins containing zinc finger domains that are involved in regulating the plant salt stress response include C2C2-type zinc finger proteins. *Arabidopsis* stress-associated RNA-binding protein 1 (SRP1) contains three C2C2-type zinc finger motifs. Its expression is downregulated by both ABA and salt stress [45]. When SRP1 is knocked out, the sensitivity of seeds to ABA is reduced, and salt tolerance is enhanced during the germination and post-germinative growth stages. Conversely, overexpressing SRP1 increased the sensitivity to both ABA and salt [45]. ABSCISIC ACID-INSENSITIVE 2 (ABI2) is an active protein phosphatase 2C (PP2C) known to play a crucial role in salt stress and ABA responses. In vitro analysis reveals that AtSRP1 binds to the 3′ UTR of the *ABI2* mRNA. The binding enables AtSRP1 to regulate the mRNA stability of *ABI2*, thereby influencing its expression and the ABA signaling pathway associated with the salt stress response [45]. Similarly, another TZF protein, the oxidation-related zinc finger protein 2 (AtOZF2), was found to be localized on the cytoplasmic membrane and can respond to salt stress through the ABI2-mediated signaling pathway [150].

Collectively, zinc finger proteins, with their diverse classifications and complex structural features, clearly play multifaceted roles in regulating plant responses to salt stress. The TZF family, despite significant progress in understanding their basic characteristics and some functional implications, still presents numerous unanswered questions regarding their precise molecular mechanisms. The involvement of other zinc finger proteins like SRP1 and AtOZF2 further highlights the complexity of the regulatory networks at play. Future research should focus on elucidating the sequence-specific functions of these proteins, identifying their in vivo target RNAs and clarifying the full extent of their interactions within the intricate signaling pathways related to salt stress.

### 3.4. DEAD-Box RNA Helicases (DBRHs)

RNA helicases (RHs) are a diverse class of ATP-dependent enzymes. Their primary function lies in the unwinding of RNA double strands and the remodeling of RNP structures [151]. Of the six RH superfamilies (SFs), the most predominant is the SF2 superfamily [152]. The SF2 proteins are further divided into three subfamilies, namely, the Asp-Glu-Ala-Asp (DEAD)-box, Asp-Glu-Ala-His (DEAH)-box, and DExD/H-box, based on sequence variations within the conserved motif II (helicase signature sequence) [153]. Among them, DEAD-box RNA helicases (DBRHs) comprise the largest subgroup, which are ubiquitously distributed across prokaryotic and eukaryotic kingdoms. Structural analysis reveals that DBRHs contain two RecA-like domains harboring conserved motifs essential for ATP-dependent RNA binding and hydrolysis-driven conformational changes, enabling RNA strand separation [154]. These enzymes participate in nearly all RNA metabolic processes including transcription elongation, pre-mRNA splicing, nucleocytoplasmic transport, and RNA turnover [155].

In plant cells, DBRHs have diverse subcellular distributions. Two *Arabidopsis* DBRHs, stress response suppressors 1 and 2 (AtSTRS1/2), exhibit dynamic nuclear–cytoplasmic shuttling during salt stress and ABA signaling [83,84]. Their expression is markedly downregulated by salt stress while loss-of-function *strs* mutants demonstrate increased salt tolerance [83,84]. The expression of several genes encoding stress-responsive proteins, such as dehydration response element-binding protein (DREB) DREB1A and DREB2A, and their downstream target gene RD29A, are enhanced in *strs* mutants, suggesting that AtSTRS1/2 plays a negative regulatory role in the activation of salt-responsive genes [83,84]. Another DBRH member, AtRH17, is exclusively localized to the *Arabidopsis* nucleus. The nuclear localization contributed to its function in modulating the expression of nine stress-responsive genes including *DREB*, *RD29A*, *RAB18*, and *RD22*. Transgenic lines overexpressing *AtRH17* exhibit improved survival under salt stress conditions. Interestingly, AtRH17 has orthologs but no paralogs in other species, which indicates that AtRH17 plays a unique role in *Arabidopsis* [85].

*Arabidopsis Osmotic Response Gene 4 (LOS4)* encodes DBRH protein AtRH38. AtRH38 is highly enriched around the nuclear membrane, and its core function is to participate in the regulation of nucleocytoplasmic transport and stability of mRNAs [37]. *Brassica napus BnRH6* is a homologous gene of *LOS4*. Its overexpression leads to a salt-sensitive phenotype [86]. Unlike AtRH38, BnRH6 is targeted to the nucleus and cytoplasmic processing body (P-body), suggesting a role for BnRH6 distinct from LOS4. Transcriptomic analysis revealed that most of the genes regulated by BnRH6 are transcription factors, ABA-responsive genes, and detoxified components or antioxidants.

In tomato, the overexpression of SlDEAD31 significantly enhances the plant tolerance to salt stress. Notably, while SlDEAD31 is widely expressed in various tissues of tomatoes, such as root, stem, leaf, and flower tissues, its expression in roots is significantly upregulated under salt stress. Moreover, SlDEAD31 enhances the plant’s salt tolerance by participating in the expression of salt stress-related genes such as the ethylene response factor *ERF1* and the pathogenesis-related protein genes *PR1/PR5* [93]. In addition to stress-related genes, those related to the process of photosynthesis in chloroplasts are also regulated.

Relatively speaking, DBRHs are more actively involved in RNA metabolism within chloroplasts and mitochondria, which will be summarized later in this article. These functions together ensure the cell’s ability to respond to environmental changes and enhance the plant’s tolerance to abiotic stresses. To date, although some DBRHs have been demonstrated to participate in regulating plant abiotic stress responses, particularly in response to temperature, the majority of DBRH family members remain unexplored, especially those associated with the response to salt stress.

### 3.5. KH Domain-Containing Proteins

The KH domain, typically composed of an evolutionarily conserved sequence of around 70 amino acids characterized by a critical signature sequence, (I/L/V)IGXXGXX(I/L/V) [156], ranks as the second most prevalent RNA-binding domain, following the RRM. The KH domain-containing proteins can specifically recognize and bind to single-stranded RNA molecules, thereby exerting regulatory effects on transcriptional and post-transcriptional gene expression processes [156].

In the plant kingdom, KH proteins play a pivotal role in multiple aspects, including RNA metabolism, flowering regulation, and stress responses. Their functions are especially prominent in flower development. For instance, the KH proteins, FLK and PEP, influence the development of floral organs by modulating the post-transcriptional modification of MADS-box genes [157,158]. However, our understanding of their functions in plant abiotic stress responses remains severely limited, and much of the available research is on AtHOS5. Under salt stress conditions, AtHOS5 engages in an interaction with FIERY2 (FRY2)/C-TERMINAL DOMAIN PHOSPHATASE-LIKE 1 (CPL1) within nuclear speckles, leading to the formation of a functional complex [106]. The plant FRY2 is an RBP and is characterized by its two dsRNA-binding domains. Its mutation significantly upregulates the expression of stress-responsive genes, including *RD29A*, *COR15A*, *KIN1*, and *COR47*, leading to an enhanced tolerance to salt stress specifically during the seed germination stage [107]. The physical interaction of AtHOS5 with FRY2 enables the resultant complex to actively participate in the splicing process of precursor mRNA, especially in crucial 5′-end capping and transcript stabilization steps [106]. Through these actions, it exerts a regulatory influence on the expression of genes associated with salt stress responses.

### 3.6. PUM Proteins

PUM proteins usually contain a C-terminal PUF domain that is highly conserved and has the ability to bind to mRNA molecules. This domain is composed of eight imperfect tandem repeats, each approximately 36 amino acids (AAs) in length, forming a curved three-dimensional structure [42]. The PUF domain has the ability to recognize a PUM-binding element (PBE), which is an eight-nucleotide conserved motif UGUAHAUW. The RNA motif is predominantly located in the 3′UTR of target mRNAs [159]. A previous study based on an analysis with Plaza 2.5 has revealed that the genomes of *Arabidopsis*, rice, soybean, apple, and corn each encode over 20 putative PUM homolog proteins, a number higher than that in most green algae [160]. The relatively large number of PUM proteins in higher plants implies their potential involvement in processes related to coping with both abiotic stresses stemming from adverse environmental conditions and biotic stresses resulting from pathogen infections [160].

The *Arabidopsis* APUM23 has been reported to respond to salt stress. It localizes in the nucleus and participates in the processing of 35S rRNA, as well as unprocessed 18S and 5.8S poly(A) rRNA within the nucleolar region of *Arabidopsis* [161]. Double mutations of *apum23* and its allele *salt-hypersensitive (sahy)* result in a phenotype that is highly sensitive to salt stress [74]. Apart from its potential role in regulating the expression of genes related to ribosome biogenesis, APUM23 downregulates the expression of the genes associated with ABA biosynthesis and signaling [74]. Notably, under salt stress, the expression levels of key ABA-responsive marker genes such as *NCED3*, *ABI2*, and *PP2CA* were reduced in the mutant accompanied by lower ABA contents. This relationship is further substantiated by the finding that exogenous application of ABA rescues the salt-hypersensitive phenotype in the *sahy9/apum23* mutant [74].

PUM proteins display diverse and dynamic subcellular localization patterns [162]. With the exception of the nuclear-localized APUM23, PUM proteins perform distinct functions in other intracellular compartments [163]. For instance, APUM5 is localized in multiple sites including the nucleus, cytoplasm, plasma membrane, and tonoplast [163]. It exerts a negative regulatory effect on the expression of the salt stress-responsive gene *RD22*, acting at both the mRNA and protein levels by binding to its 3′ untranslated region [73]. Transgenic plants overexpressing APUM5 exhibit robust tolerance to salt stress [73]. Another investigation [75] reported that APUM6, with detectable RNA-binding activity, is situated on the surface of the endoplasmic reticulum (ER). This finding implies that APUM6 might function as a post-translational regulator with specific target molecules unrelated to stress-related gene expression [75]. Interestingly, the silencing of APUM6 leads to salt tolerance, albeit accompanied by a decrease in the seed germination rate. The diverse localization of APUM proteins is manifested by the fact that plant PUMs may shuttle between the cytoplasm and the nucleus. It is reported that APUM5 contains a putative transmembrane domain and exhibits cytoplasmic discontinuity structures when observed in tobacco epidermal cells [164,165]. Additionally, the localization of APUM23 in nucleoporins and the discovery of APUM6 within stress granule structures [75] further support this notion. These studies on dynamic localization strongly imply that PUM proteins are involved in various transport processes from the nucleus to distinct intracellular locations within the cell.

### 3.7. RBPs Involved in Cytoplasmic RNA Granule Formation

Under salt stress, the equilibrium of RNA metabolism encompasses multiple processes such as RNA decay, translation, and sequestration. Translational control involves two key events: the formation of mRNP complexes and the assembly of membrane-less RNA granules via liquid–liquid phase separation (LLPS) [166]. Stress granules (SGs) are dynamic cytoplasmic condensates formed under adverse conditions. These structures rely on LLPS to aggregate translationally repressed mRNAs and associated proteins, temporarily halting translation while protecting mRNAs from degradation. SGs are reversible condensates that can be disassembled upon stress alleviation [166]. Functionally, SGs are closely associated with processing bodies (P-bodies) [167]. P-bodies contains the key factors for mRNA decay including decapping proteins (DCPs), 5′ to 3′ exoribonuclease, de-adenylation factors, small-RNA-dependent slicer protein Argonaute 1, and factors involved in nonsense-mediated mRNA decay [168]. Those factors govern mRNA degradation including deadenylation, mRNA decapping, 5′ to 3′ degradation, and co-translational decay [169]. The connection between SGs and P-bodies provides the basis for the close relationship between mRNA decay and translational repression.

RBPs play a central role in SG formation. In animals, T-cell intracellular antigen 1 (TIA-1) and TIA-1-related (TIAR) proteins act as nucleation factors for SG assembly under nutrient/energy stress [170,171]. Higher plants have evolved analogous systems that are executed mainly by RBPs including oligouridylate-binding protein 1 (UBP1), RNA-binding proteins 45 and 47 (RBP45/47), and the Poly(A)-binding (PAB) protein families [172]. All of these RBPs possess triple RRMs, in which UBP1 shares the highest sequence similarity with animal TIA1/Rs. The UBP1 family in *Arabidopsis* contains three members, namely, UBP1a/b/c [146]. While all the three UBP1 proteins participate in SG formation [173], UBP1c specifically binds U-rich 3′UTRs, with expanded binding repertoire under hypoxia [172]. UBP1b is reported to enhance the stability of the RNAs of a DnaJ heat shock protein and a stress-associated protein (AtSAP3) through SG formation, which directly contribute to the enhanced heat tolerance in UBP1b overexpression lines [173]. On the other hand, *ubp1b* mutants are reported to be sensitive to salt and osmotic stress [105]. Given its demonstrated RNA-binding capabilities and its role in SG-related processes under different conditions, it is reasonable to assume that UBP1 may also be a promising candidate for future studies on stress granule dynamics in plant salt tolerance.

Tudor staphylococcal nuclease (TSN) family proteins are evolutionarily conserved RBPs [174] with dual roles in RNA metabolism and SG dynamics. In *Arabidopsis*, two redundant paralogs (TSN1 and TSN2) have been identified [109]. Under normal growth conditions, AtTSN1 shows diffuse cytoplasmic localization. However, when exposed to salt stress, it translocates into cytoplasmic SGs and co-localizes with the SG marker protein RBP47 [109]. It has been reported that TSN promotes plant growth under salt stress by binding to and stabilizing mRNAs encoding secretory pathway components, such as GA20ox3, which is involved in GA biosynthesis [109]. Loss of TSN function results in accelerated mRNA degradation and reduced salt tolerance [109,175]. These findings highlight TSN’s role as a translational regulator that balances stress responses and developmental programs.

Recent study has uncovered a functional partnership between TSN1 and RH31, a salt-responsive DBRH protein [87]. Under salt stress, RH31 binds to and stabilizes stress-responsive mRNAs, such as *AREB1*, *P5CS1*, and *RD29B*, and then translocates from the nucleus to SGs in a TSN1-dependent manner. In the process, TSN1 enhances RH31 helicase activity and promotes SG formation [87]. This interaction establishes a regulatory axis where TSN-RH complex coordinates mRNA stabilization and translational control during salt stress.

Certain RBPs exhibit dual localization in both P-bodies and SGs, enabling coordinated responses to salt stress. In mammals, tandem zinc finger proteins (TZFs) act as nucleocytoplasmic shuttling factors critical for the assembly of both P-bodies and SGs. Functional conservation of this mechanism exists in plants. Representative examples are AtTZFs and OsTZFs that have been shown to be localized in PBs and SGs in *Arabidopsis* and rice cells, respectively [148]. A recent study on the previously aforementioned AtTZF1 [176] found that this protein undergoes stress-induced re-localization from the nucleus to SGs, where it downregulates vacuolar calcium pumps (ACA11/ACA4) to maintain cellular homeostasis [77]. In rice, OsTZF1 demonstrates dynamic subcellular distributions. The protein exhibits nucleocytoplasmic shuttling under normal conditions but accumulates in both P-bodies and SGs under salt stress. The stress-specific localization in P-bodies and SGs is demonstrated by co-localization with DCP2 and PABP8, respectively. Its RNAi lines show severe leaf damage and mortality under salt treatment, while its overexpression confers salt tolerance [78]. This functional similarity to human tristetraprolin (TTP) suggests conserved regulatory mechanisms across kingdoms [177]. This indicates that OsTZF1 is likely to dynamically participate in the assembly of SGs under stress. Similar functional conservation is observed in other plant species. For instance, HuTZF3 from pitaya (*Hylocereus polyrhizus*) co-localizes with both P-body and SG markers with expression patterns supporting stress tolerance [81]. These findings highlight the abilities of TZF proteins to dynamically partition between P-bodies and SGs, which allows for the integrated control of mRNA decay and translational repression under salt stress. Future studies should focus on elucidating the precise molecular mechanisms underlying their RNA target selection and spatiotemporal regulation.

YT521-B homology (YTH) domain family proteins are recognized for their role as direct N6-methyladenosine (m6A) readers, exhibiting a strong binding affinity for m6A-modified mRNA. *Arabidopsis* EVOLUTIONARILY CONSERVED C-TERMINAL REGION 8 (ECT8), a member of the YTH protein family, has been reported to regulate mRNA decay [82]. This protein is localized in the cytoplasm. Under salt stress conditions, both the transcription level and protein abundance of ECT8 increase. Concurrently, its binding capacity to m6A-modified mRNAs is also enhanced [82]. The binding enables ECT8 to accelerate the degradation of m6A-modified mRNAs, which is facilitated by directly interacting with DCP5 within P-bodies [82]. Mutation of ECT8 results in increased expression levels of negative regulators of salt stress, leading to elevated sensitivity to salinity as manifested by delayed germination, reduced growth rate of green cotyledons, shorter root length, and a shorter main stem length [82]. Thus, ECT8 may serve as an abiotic stress sensor by accelerating mRNA decay of negative regulators of salt stress to maintain transcriptome homeostasis and stress tolerance. Interestingly, another study has demonstrated that, upon treatment with a high concentration of ABA, ECT8 translocates to SGs, leading to the inhibition of protein translation [178]. It is known that, aside from the similarities between the formation processes of P-bodies and SGs, certain components of P-bodies can dynamically shuttle between P-bodies and SGs under stress conditions. In other words, P-bodies and SGs can coexist under conditions such as salt stress [179]. This suggests that ECT8 may possess a dual function in promoting mRNA degradation in P-bodies and halting mRNA translation in SGs under stress. On the other hand, the heterologous expression of MhYTP1 or MhYTP2, which are members of YTH family proteins from *Malus hupehensis* (Pamp.) Rehd, has been found to enhance salt tolerance in *Arabidopsis* [39]. This further confirms the function conservation among YTH family proteins and their indispensable role in the post-transcriptional regulation of plants under salt stress.

It should be noted that, although some RBPs have been identified to translocate from the nucleus to the cytoplasm in response to salt stress and participate in the formation of SGs, the variety of RBP types investigated thus far is rather limited. Additionally, for those RBPs present in both P-bodies and SGs, research into their downstream gene networks is still in its infancy and requires further in-depth exploration.

### 3.8. Other Salt-Responsive RBPs Localized to the Nucleus and Cytoplasm

Another nucleus-localized RBP functionally characterized for its role in salt stress tolerance is AlSRG1, an RBP harboring a single RRM domain, identified from *Aerospora littoralis*. Transgenic tobacco overexpressing *AlSRG1* displays enhanced seed germination under salinity, primarily through its ability to transcriptionally upregulate ROS-scavenging enzymes and stress-responsive genes, thereby maintaining cellular redox homeostasis [104]. This study further demonstrates the participation of RRM domain-containing proteins in plant salt adaptation.

The cytoplasm-localized AtRGGA contains a Suppressor of Tom1 (Stm1) domain and a Hyaluronan-Binding Protein 4_Plasminogen Activator Inhibitor-1 mRNA-Binding Protein 1 (HABP4_PAI-RBP1) domain. The two domains were previously found in yeast Stm1 nucleic acid-binding protein and certain RBPs, respectively. AtRGGA binds to RNA in a highly conserved manner, relying on the HABP4-PAI-RBP1 domain within its structural composition [108]. It has been found that AtRGGA localizes in the vicinity of the cytoplasm and the nucleus. Its expression can be induced by ABA exposure but is downregulated under salt treatment [108]. Overexpression of *AtRGGA* can enhance the nucleocytoplasmic transport efficiency of stress-responsive genes like *RD29A*, while simultaneously inhibiting the nuclear export of mRNAs related to proline synthesis [108,180]. The study suggests that AtRGGA has the ability to regulate the stability or translation efficiency of mRNA under salt stress conditions.

SOAR1, a member of the PPR family of proteins, is another protein that directly responds to salt stress by regulating the ABA signaling pathway. Different from other members of the family, it is localized in both the nucleus and the cytoplasm and serves as a key regulatory factor in the ABA signal transduction pathway [98]. SOAR1 can influence the translation of target mRNAs (such as *ABI5*) by binding to them, thereby regulating the ABA signaling pathway and directly influencing the plant’s response to salt stress. Overexpression of *AtSOAR1* in *Arabidopsis* leads to higher survival rates and enhanced seed germination under salt stress conditions [98].

Another notable RBP is AGO1, a member of the highly conserved Argonaute family. Argonaute proteins typically interact with small RNAs such as microRNAs (miRNAs) and small interfering RNAs (siRNAs) through its (Piwi/Argonaute/Zwille) PAZ domain and act as a slicer for miRNA binding and cleavage to regulate gene expression, which mainly occurs in RNA-induced silencing complex (RISC) complex [181]. Recent studies have revealed a unique regulatory mechanism in rice chloroplasts, in which microRNA168 (miR168) directly targets OsAGO1 and thus regulates its transcriptional activation [110].

Collectively, all the abovementioned RBPs localized in nucleus and cytoplasm primarily carry out functions such as RNA splicing, maintaining mRNA stability, RNA granule formation, and facilitating the export of mRNA from the nucleus, yet they may also participate in transcriptional activation. However, the precise mechanisms by which they regulate target RNAs, as well as the identity and characteristics of the target RNAs they bind to, remain largely unknown and await further exploration.

## 4. RBPs in Chloroplasts and Mitochondria

Plant cell chloroplasts and mitochondria, serving as pivotal organelles for photosynthesis, energy conversion, and substance synthesis, are not only crucial for plant survival under normal growth conditions; they also play an indispensable role in the plant’s response to salt stress. Chloroplasts and mitochondria evolved from endosymbiotic bacteria, and during evolution, there is extensive gene transfer from plastids to the nucleus [182]. As a result, many nuclear-encoded proteins that target chloroplasts and mitochondria are fundamental for regulating gene expression within both organelles. In the context of adapting to environmental stress and throughout the processes of plant growth and development, the regulation of post-transcriptional RNA processing within these organelles relies on hundreds of nuclear-encoded chloroplasts or mitochondrial RNA-binding proteins (nCMRBPs) [183]. In recent years, nCMRBPs have increasingly attracted research attention. A wealth of studies has indicated that they are vital for the fine-tuned regulation of post-transcriptional RNA metabolism in plant mitochondria and chloroplasts during salt stress responses. This regulation encompasses various aspects, including RNA processing, intron splicing, RNA stability, editing, and translational control [184]. An analysis of the characteristics of nCMRBPs reveals that they possess multiple conserved motifs and domains. Their core members consist of four major families: chloroplast ribosome maturation and splicing domain (CRM), S1-domain-containing protein (SDP), DBRH, and PPR [111,142,185,186]. These proteins, which localize to either chloroplasts or mitochondria, interact cooperatively or independently to form a complex regulatory network. The following section focuses on presenting the recent research advancements regarding the functions and cellular mechanisms of these four protein families in the RNA metabolism of chloroplasts and mitochondria during the response to salt stress.

The CRM domain was initially identified in archaea and bacteria and shares orthology with the YhbY protein of *Escherichia coli* [187]. A distinctive characteristic of this domain is the conserved GxxG sequence (glycine-X-X-glycine), which is located in the loop region of the domain. This sequence can directly engage in RNA binding [186,187,188]. In land plants, through gene duplication and functional divergence, four major subfamilies have emerged within the CRM protein family. These are chloroplast RNA splicing 1 (CRS1), the CAF subfamily (also referred to as CRS2-associated factors), CRM family member 3 (CFM3) and CFM4 [189]. As a crucial factor in RNA splicing, the CRM domain plays a vital role in the RNA metabolism of plant organelles, predominantly chloroplasts and mitochondria [190]. This, in turn, has a profound impact on the regulation of plant growth and development. Loss-of-function mutations in CRM proteins can result in severe phenotypic abnormalities, such as abnormal chloroplast development [191] and mitochondrial functional deficiencies [192] as well as accelerated seed germination and plant senescence processes [96]. Moreover, certain CRM proteins facilitate plant adaptation to temperature and salt stress by modulating RNA metabolism. For instance, *Arabidopsis* CFM4 is reported to exert a positive influence on seed germination and seedling growth of *Arabidopsis* under salt stress conditions. CFM4 acts as an RNA chaperone and is involved in the processing and maturation of 16S and 4.5S rRNA within the chloroplast. This finding is consistent with the fact that prokaryotic proteins with a single CRM domain participate in ribosome maturation [96]. Owing to this function, T-DNA insertion mutants of *cfm4* manifest a series of phenotypic alterations. Under normal growth conditions, these mutants exhibit retarded growth and a delay in senescence. When subjected to stress conditions such as salt and cold stress, they show exhibit inhibited seed germination and seedling growth [96]. These observations imply that CFM4 plays a vital role in the plant growth, development, and stress response.

The S1 domain is widely dispersed across both prokaryotes and eukaryotes and represents a conserved RNA-binding module. Its nomenclature is derived from the ribosomal protein S1 in bacteria. A core characteristic of the S1 domain is the presence of the OB-fold (oligonucleotide/oligosaccharide-binding fold) structure. This structure contributes to the specific recognition of single-stranded RNA (ssRNA) via a β-barrel architecture [41]. Most of the reported plant S1 domain-containing proteins (SDPs) are directed to chloroplasts. These proteins interact with ribosomal *23S*, *16S*, *5S*, and *4.5S* RNAs [193]. This interaction enables them to tightly engage with the chloroplast ribosomal RNAs and play a pivotal role in chloroplast gene expression, stress response mechanisms, and the regulation of plant development [194]. In the case of *Arabidopsis*, similar to CFM4, a chloroplast-targeted SDP exhibits RNA chaperone activity. It can bind to and participate in the processing of chloroplast 16S, 23S, 4.5S, and 5S rRNAs [111]. In the *sdp* mutant, the impaired rRNA processing within the chloroplast leads to a reduction in chlorophyll a content and photosynthetic activity. Consequently, this results in stunted growth, delayed seed germination, and pale-green phenotypes [111]. A subsequent study disclosed that the loss of SDP function causes a decline in the survival rate of *Arabidopsis* under various stresses, including salt, heat, UV, and freezing stresses but not dehydration stress [112]. These findings emphasize that SDP, like other nCMRBPs like CFM4, is an essential component in the intricate network that allows plants to maintain proper chloroplast function and to adapt to various stress conditions, particularly salt stress, through its role in rRNA processing and the associated physiological effects on plant growth and survival.

As previously mentioned, certain DBRH family proteins are targeted to chloroplasts. Some of them are also involved in rRNA processing and, consequently, ribosome biogenesis. For example, AtRH22 is reported to regulate the assembly of the 50S ribosomal subunit via rRNA processing, which ensures the normal operation of the chloroplast translation machinery [195]. The *Brassica oleracea* BrRH22, the ortholog of AtRH22, can also participate in the assembly of the 50S ribosome. Additionally, the RNA chaperone activity of BrRH22 enables it to influence the translation of chloroplast transcripts *PSBA*, *RBCL,* and *YCF3*. Collectively, these properties contribute to enhanced salt tolerance, as manifested by increased seed germination and improved plant growth under high salinity, by *Arabidopsis* heterologously expressing BrRH22 [91]. However, this phenotype is exactly opposite to that of its homolog in rice, OsRH58. OsRH58 has the ability to bind to a variety of chloroplast mRNAs and remodels their structure, thereby affecting the translation of several target mRNAs including *POR*, *RbcL*, *ClpB3*, *PsbA,* and *PetA* [92]. In barley, a chloroplast-localized DEAD-box protein, Hordeum vulgare DEAD-box protein (HVD1) is typically expressed at low levels in leaves and roots but is significantly upregulated under salt stress. Although the detailed mechanism remains largely unknown, it is proposed that HVD1 affects photosynthetic activity by upregulating the expression of the relevant genes at the RNA level [89]. Furthermore, some chloroplast-targeted DBRH family proteins function as RNA chaperones during the RNA splicing process within the chloroplast under salt stress conditions. For instance, in *AtRH3* mutant plants, the splicing of several intron-containing chloroplast genes, including *NADH dehydrogenase subunit A* (*ndhA*) and *B* (*ndhB*), were found to be defective [88]. AtRH3 likely facilitates the proper folding of intron-containing RNAs in chloroplasts, enabling the formation of catalytically active structures within the organelle. The function of RH3 likely exhibits cross-species functional conservation, as the overexpression of *GmRH3* in soybean significantly enhances the salt tolerance of soybean plants, as demonstrated by increased root elongation and biomass [90].

The PPR protein family represents one of the largest gene families in land plants. Typically, each species encodes over 400 members; for instance, *Arabidopsis* has approximately 500 PPR protein-encoding genes, and tomato has 471 [142]. Encoded by the nuclear genome, these proteins localize primarily to mitochondria and chloroplasts. PPR proteins are characterized by the presence of tandem 35-amino acid repeat motifs arranged in a solenoid-like structure. Each PPR motif forms a helix–turn–helix fold, and the entire array creates a platform for sequence-specific RNA recognition through critical amino acid residues [196]. This structural feature enables PPR proteins to play central roles in organellar RNA metabolism, regulating processes such as splicing, processing, stability, and translation [197]. Functional diversification within the PPR family is accomplished through variations in the C-terminal domain variations [197]. The P subfamily, which is defined by canonical PPR motifs, mainly mediates RNA stability, processing, and translational regulation. In contrast, the PLS subfamily, which is identified by extended PPR motif-like sequence (PLS) repeats and appended E/E+/DYW domains, specializes in RNA editing [38,198]. Due to their roles in RNA metabolism, PPR proteins influence plant development and stress responses by modulating photosynthesis- and respiration-related gene expression. Recent evidence also highlights their involvement in mitochondrial reactive oxygen species (ROS) homeostasis, linking them to disease resistance and salt stress adaptation [103]. Additionally, PPR proteins have been implicated in male sterility and fertility restoration processes in crops such as rice, maize, and *Arabidopsis* [199].

In the context of salt stress, one of the chloroplast-localized PPRs is rice WSL, which is named based on its white stripe leaf mutant phenotype. WSL belongs to the PLS subgroup and contains 14 PPR-like motifs but is devoid of the typical E/E+ or DYW domain in the C-terminus. Its mutation results in a low content of chlorophyll and carotenoid in leaves and serious damage of chloroplast structure [99]. In addition, the *wsl* mutant exhibits hypersensitivity to ABA and salt stress in which the enhanced sensitivity to salt is exclusively detected at the seed germination stage [99]. Severe reductions in chloroplast rRNAs and chloroplast-encoded photosynthetic proteins are observed in the *wsl* mutant suggesting an essential role of WSL in the biogenesis of ribosomal subunits and the photosynthetic complex. Unlike other PPR proteins, WSL does not possess the ability to edit RNA but, instead, is involved in RNA splicing of chloroplast genes. One of the target genes is *rpl2* [99]. The *rpl2* gene encodes the ribosomal protein subunit L2 (RPL2), which is an essential element of the chloroplast translation apparatus. In the *wsl* mutant, splicing of *rpl2* transcripts is impaired resulting in aberrant *rpl2* transcript accumulation and, in turn, a reduction in RPL2 protein [99]. While these findings shed light on the complex interplay between PPR proteins and chloroplast biogenesis, future research could focus on exploring the upstream regulatory factors of WSL and the specific molecular interactions it engages in.

Mitochondria-localized PPRs associated with salt stress are exemplified by *Arabidopsis* PPR40, PGN, and PPR96. PPR40 mediates adaptive responses to salt stress through integrated regulation of redox homeostasis and energy metabolism. The *ppr40* mutant exhibits pleiotropic phenotypes such as delayed germination, semi-dwarfism, and hypersensitivity to NaCl. These phenotypes are correlated with disrupted mitochondrial electron transport chain (ETC) activity and a concomitant redox imbalance, resulting in the over-accumulation of ROS and a reduction in antioxidative capacity [100,101]. Functional analysis has revealed that PPR40 maintains Complex III integrity in the mitochondrial ETC, thereby sustaining the continuous production of ATP and proper redox signaling under salt stress conditions. Transgenic lines overexpressing PPR40 display enhanced seed germination rates, elevated SOD/CAT/APX activities, and reduced MDA accumulation, indicating an improved ability to salvage ROS [100]. Notably, PPR40 physically interacts with Complex III subunits, suggesting a direct role in maintaining respiratory chain efficiency during osmotic stress [101]. Beyond redox regulation, PPR40 modulates ABA signaling by upregulating ABA-responsive genes such as *RD29A* and *COR15A*, while simultaneously downregulating the ABA catabolic enzyme CYP707A2. This dual regulation helps to reduce ROS-mediated damage by promoting stomatal closure and enhancing stress-responsive gene expression. Intriguingly, *ppr40* mutants exhibit a high basal expression of another PPR gene, *PENTATRICOPEPTIDE REPEAT PROTEIN FOR GERMINATION ON NaCl (PGN),* and correspondingly, *pgn* mutants exhibit a phenotype similar to that of the *ppr40* mutant. These findings suggest that PPR40 may regulate the transcription of PGN and that there are mutual interactions among various PPRs.

It is interesting to note that the high basal expression of PGN is repressed during *Botrytis cinerea* infection, suggesting crosstalk between mitochondrial function and plant immunity. The observed transcriptional dysregulation of PPR may stem from retrograde signaling defects, as mitochondrial dysfunction often perturbs nuclear gene expression via ROS and metabolite signaling pathways. It is worth mentioning that PGN has a critical regulatory role in the expression of mitochondrially encoded genes *AOX1*, *NAD1*, *RPL2*, *NAD9,* and *MATR* [95]. Similarly, PPR96, a mitochondrially localized ortholog of PPR40, plays a conserved role in salt stress adaptation. In *Arabidopsis*, its loss-of-function mutation results in a similar pleiotropic phenotype. In all these cases, PPR likely coordinates mitochondrial RNA editing events essential for maintaining respiratory chain integrity under osmotic stress. However, the exact RNA targets and functional consequences of PPR96 remain to be explored [102].

A previous study revealed that the expression of *AtRH9* and *AtRH25* were repressed under salt stress [84]. Phenotypic analysis of transgenic *Arabidopsis* overexpressing *AtRH9* or *AtRH25* exhibited a lower germination rate under stress conditions. This suggests that AtRH9 and AtRH25 may be negative regulators of salt stress tolerance. Additionally, an in vitro assay showed that AtRH9 binds equally to all homoribopolymers, whereas AtRH25 prefers binding to poly(G) [84], suggesting a significant difference between the nucleic acid-binding activities of the two RHs. While AtRH9 and AtRH25 are proposed to regulate mRNA metabolism [84], AtRH9 is predicted to localize in the mitochondria and act as a potential antioxidant protein [200].

It has been reported that the expression of DBRH can be regulated microRNAs [94]. For example, rice *OsABP* (*ATP-Binding Protein*), *OsDSHCT* (*DOB1/SK12/helY-like DEAD-box Helicase*) and *OsDBH* (*DEAD-Box Helicase*) genes are reported to be the target gene of *osa-MIR414*, *osa-MIR408,* and *osa-MIR164e* [94]. While the expression of *osa-MIR414*, *osa-MIR408,* and *osa-MIR164e* is downregulated by salt stress treatment, the expression of the three *DBRH* genes are upregulated in response to 100 and 200 mM NaCl treatments. The negative correlation between the miRNAs and DBRH genes suggests that miRNAs likely play a role in post-transcriptional regulation of DBRH genes under salt stress. This regulatory mechanism may be part of the plant’s adaptive response to salt stress.

Rice Suppressor of Var 3 (OsSUV3) is a mitochondrial-localized DExH/D type RNA helicase [201]. Its expression is induced by high salinity stress [95,202]. When compared to wildtype, the overexpression of OsSUV3 confers salinity tolerance. This is accompanied with lesser lipid peroxidation, electrolyte leakage, and H_2_O_2_ production, along with higher activity levels of antioxidant enzymes under salinity stress [95,202]. The results of this study suggest that OsSUV3 likely enhances salinity stress tolerance by maintaining photosynthesis and antioxidant machinery in rice. Further analysis has revealed that OsSUV3 is localized in the mitochondria and is a component of the mitochondrial degradosome complex [95,202]. It can participate in the overall cellular homeostasis and gene expression by regulating the transcription and degradation of RNA in the mitochondria [95,202]. Specifically, it can restore the native conformation of some misfolded RNAs under salt stress.

Another nCMRBP involved in mitochondrial RNA metabolism is CFM9. Similar to the chloroplast CFM4, CFM9 also contains only a single CRM domain. Notably, while chloroplast CFM4 participates in rRNA processing in chloroplasts [96], CFM9 has a distinct function in the splicing of many intron-containing mitochondrial RNAs, thereby influencing the generation of mature transcripts such as those of *nad1*, *nad2*, and *nad4*. In the context of mitochondrial functions related to plant development, CFM9 plays a positive role in both seed germination and seedling growth. This positive effect is more pronounced under high salt stress conditions. Simultaneously, high salt stress also leads to an impact on root growth, where CFM9 likely contributes to the overall stress response mechanisms in the plant’s root system [97].

Collectively, nCMRBPs that function as RNA chaperones hold a pivotal position in the intricate biological processes within plants. During the plant’s resistance to salt stress, these nCMRBPs are actively involved in the regulation of mitochondrial and chloroplast RNA metabolism. They ensure the proper processing of RNA transcripts, which is crucial for the synthesis of essential proteins required for chloroplast and mitochondria RNA translation, photosynthesis, respiratory chain maintenance, energy production, redox balance, and other vital metabolic pathways.

## 5. Conclusions and Future Directions

The spatiotemporal orchestration of RNA fate by RNA-binding proteins (RBPs) across subcellular compartments represents a critical node in plant adaptive responses to salt stress. Current evidence highlights RBPs as multifunctional regulators coordinating nucleus RNA splicing, RNA stability, cytoplasmic RNA granule dynamics, and organellar RNA metabolism in chloroplasts and mitochondria. However, mechanistic insights into RBP-mediated stress responses remain fragmented, particularly regarding RNA target specificity across organelles, post-translational modifications (PTMs) modulating RBP activity, and inter-organellar communication networks.

Advancing this field requires integrating emerging technologies to address these gaps. Technical innovations including photoactivatable ribonucleoside-enhanced crosslinking and immunoprecipitation (PAR-CLIP) combined with nanopore sequencing will enable full-length RNA interactome mapping, complemented by organellar CRISPR interference (CRISPRi) screens to validate functions. Single-cell resolution approaches, such as RBP immunoprecipitation combined with single-cell RNA sequencing (scRIP-seq), could map RBP-RNA interactomes at subcellular resolution, while spatial transcriptomics would reveal dynamic targeting changes during salt acclimation. Proteomic methods including phosphoproteomics paired with CRISPR-based PTM site mutagenesis may help to dissect how phosphorylation, SUMOylation, and ubiquitination influence the functions of RBPs. Inter-organellar signaling mechanisms may be elucidated through proximity labeling (BioID) to identify novel RBP interactors in chloroplasts/mitochondria, and dual-fluorescence complementation (BiFC) assays may be used to visualize stress-induced trafficking.

Comparative analysis of RBP orthologs across various plant species using phylogenetic network reconstruction could uncover adaptive sequence motifs, informing crop improvement strategies. CRISPR-based engineering of stress-responsive RBPs holds promise for enhancing salt tolerance. Systems biology approaches integrating multiomics datasets via machine learning algorithms would predict regulatory hubs, while biophysical studies using optical tweezers or single-molecule fluorescence resonance energy transfer (FRET) could resolve RBP-RNA interaction kinetics.

These advancements not only advance fundamental plant biology but also provide targeted molecular markers for breeding salt-resistant crops. By addressing these challenges, we can establish a comprehensive framework of RBP-mediated post-transcriptional regulation, transforming our understanding of plant stress adaptation and accelerating agricultural innovation through integrative mechanistic studies and technological breakthroughs.

## Figures and Tables

**Table 1:** The RBPs involved in plant salt stress response.

RBP Types	Motif	Name	Species	Localization	Function Under Salt Stress	Ref.
GR-RBP	RRM, GR	AtGRP7	*Arabidopsis*	Nc, Cy	Regulates alternative splicing.	[46,47]
		AtGRDP2	*Arabidopsis*	Nc	Binds to the 3′UTR or the CDS of genes encoding antioxidant enzyme.	[48]
		AtGRP4	*Arabidopsis*	Nc	-	[49]
		MpGR-RBP1	*Malus prunifolia*	Nc	-	[50]
		NtGRP1	*Nicotiana tabacum*	Nc, Cy	-	[51]
		LbGRP1	*Limonium bicolor*	Nc, Cy	Regulates the Na^+^/K^+^ ratio under salt stress.	[52]
		BpGRP1	*Betula platyphylla*	Nc	-	[53]
		CsGRP2,7	Camelina (*Camelina sativa*)	Nc, Cy	-	[54]
		MhGR-RBP1	*Malus hupehensis* (Pamp.) Rehd	-	-	[55]
		SbGR-RNP	*Sorghum bicolor*	-	-	[56]
		ZjGRP	*Zoysia japonica*	Nc, Cy	Regulates the expression of salt-responsive genes.	[57]
		PeGRP2	*Populus euphratica*	-	Targets mRNAs that encode antioxidant enzymes.	[58]
		SaRBP1	*Suaeda asparagoides*	Cy	-	[59]
RZ	RRM, ZF, GR	AtRZ-1a	*Arabidopsis*	Nc, Cy	Regulates the expression of germination-responsive genes and ROS-related genes.	[60]
		BrRZ2,3	Cabbage (*Brassicarapa*)	Nc	Assist in the RNA folding process.	[61]
		TaRZ1,2,3	Wheat (*Triticum aestivum*)	Nc	-	[62]
CSDP	CSD, ZF, GR	AtCSDP2	*Arabidopsis*	-	Enhances salt tolerance during seedling establishment.	[58]
		CSP2	*Arabidopsis*	-	Stabilizes stress-responsive mRNAs by binding their 3′UTRs.	[63]
SR/RS	RRM, SR/RS-rich	AtSR45a-1a,1b	*Arabidopsis*	Nc	Bind to CBP20 and regulate alternative splicing and mRNA stability.	[64]
		AtSR45	*Arabidopsis*	Nc	-	[65]
		SR33	*Arabidopsis*	Nc	Co-assembles with the TREX complex to regulate nucleocytoplasmic transport of mRNA.	[66]
		RSZ22				
		RSZ33				
		MeSR34	Cassava (*Manihot esculenta Crantz*)	Nc	-	[65]
		MeRS40	Cassava (*Manihot esculenta Crantz*)	Nc	Regulates alternative splicing through action on the stability of the spliceosome complex.	[67]
		OsRS33	Rice	Nc	Regulate the alternative splicing events related to salt stress.	[68]
		AtSCL30a	*Arabidopsis*	Nc	-	[69]
		MeSCL30a	Cassava (*Manihot esculenta Crantz*)	Nc	-	[70]
		PtSCL30	*Populus trichocarpa*	Nc	-	[71]
		GhSCL-8	Cotton (*Gossypium spp.*)	Nc	-	[72]
PUM	PUF	APUM5	*Arabidopsis*	Nc, Cy	Binds to the 3′UTR of stress-responsive genes and regulate their expression.	[73]
		APUM23	*Arabidopsis*	Nc	Regulates the expression of genes related to ribosome biogenesis.	[74]
		APUM6	*Arabidopsis*	Cy, Er	Localizes in stress granules and respond to salt stress.	[75]
TZF	TZF, RR	AtSZF1 (AtTZF10)	*Arabidopsis*	Nc	Negatively regulates genes involved in salt stress response under salt stress.	[76]
		AtSZF2 (AtTZF11)	*Arabidopsis*			
		AtTZF1	*Arabidopsis*	Nc, Cy	Downregulates the expression of genes related to the vacuolar calcium pump under salt stress and participates in the formation of stress granules.	[77]
		OsTZF1	Rice	Cy	Co-localizes with PABP8 in stress granules.	[78]
		GhTZF1	*Gossypium hirsutum L.*	Nc	-	[79]
		AtTZF2	*Arabidopsis*	Cy	-	[80]
		AtTZF3	*Arabidopsis*			
		HuTZF3	Pitaya (*Hylocereus polyrhizus*)	Cy	Localizes in P-bodies and stress granules.	[81]
YTH	YTH	AtECT8	*Arabidopsis*	Cy	Promotes the degradation of mRNAs in P-bodies and halts the translation of mRNAs in stress granules.	[82]
		MhYTP1,2	*Malus hupehensis* (Pamp.) Rehd	-	-	[39]
DBRH	DEAD-box	AtSTRS1,2 (AtRH25)	*Arabidopsis*	Nc	Attenuates the expression of stress-responsive transcriptional activators.	[83,84]
		AtRH17	*Arabidopsis*	Nc	Regulates gene expression in response to salt stress.	[85]
		BnRH6	*Brassica napus*	Nc, Cy	Regulates salt-tolerant genes.	[86]
		AtRH31	*Arabidopsis*	Nc, Cy	Interacts with TSN1 during the formation of SGs and regulate the expression of salt-responsive genes.	[87]
		AtRH3	*Arabidopsis*	Ch	Acts as an RNA chaperone to regulate the splicing of chloroplast *ndhA* and *ndhB*.	[88]
		HVD1	Barley (*Hordeum vulgare*)	Ch	Regulates the expression of genes related to photosynthesis.	[89]
		GmRH3	Soybean (*Glycine max L.*)	-	-	[90]
		BrRH22	Cabbage (*Brassica rapa*)	Ch	Participates in ribosome processing and affects the translation of chloroplast transcripts.	[91]
		OsRH58	Rice	Ch	Acts as an RNA chaperone and regulates the translation of chloroplast mRNAs.	[92]
		SlDEAD31	Tomato (*Solanum lycopersicum*)	Ch	Regulates the expression of salt-responsive genes.	[93]
		OsABP	Rice	Ch	-	[94]
		OsSUV3	Rice	Mt	Maintains antioxidant mechanisms.	[95]
		AtRH9	*Arabidopsis*	Mt	Negatively regulates the response to salt stress.	[84]
CRM	CRM	CFM4	*Arabidopsis*	Ch	Acts as an RNA chaperone and participates in the processing and maturation of 16S and 4.5S rRNA.	[96]
		CFM9	*Arabidopsis*	Mt	Participates in the splicing of chloroplast introns and affects the formation of mature transcripts.	[97]
PPR	PPR	AtSOAR1	*Arabidopsis*	Nc, Cy	Binds to the targeted *ABI5* under salt stress to regulate gene expression.	[98]
		OsWSL	Rice	Ch	Participates in the splicing of *rpl2* under salt stress, leading to defects in chloroplast development.	[99]
		AtPPR40	*Arabidopsis*	Mt	Reduces the accumulation of ROS in mitochondria and associates with Complex III in the electron transport system.	[100,101]
		AtPPR96	*Arabidopsis*	Mt	May be involved in mitochondrial RNA editing.	[102]
		AtPGN	*Arabidopsis*	Mt	Regulates the expression of mitochondrial transcripts.	[103]
Others	RRM	AlSRG1	Tobacco (*Nicotiana tabacum L.*)	-	Regulates the expression of ROS-scavenging genes and stress response transcription factors.	[104]
		AtUBP1	*Arabidopsis*	Nc, Cy	Participates in stress granule formation	[105]
	KH	AtHOS5	*Arabidopsis*	Nc	Interacts with FRY2/CPL1 and participates in the splicing of precursor mRNA, thereby influencing the expression of genes related to salt stress.	[106]
	ZF	AtSRP1	*Arabidopsis*	Nc	Binds to the targeted *ABI2*.	[45]
	DSRM	FRY2	*Arabidopsis*	Nc	Regulates the expression of salt-responsive genes.	[107]
	HABP4-PAI-RBP1	AtRGGA	*Arabidopsis*	Cy	Regulates the stability or translation efficiency of mRNA.	[108]
	Tudor, SN	AtTSN	*Arabidopsis*	Cy	Participates in the assembly of stress granules and regulates the stability of mRNA.	[87,109]
	PAZ	OsAGO1	Rice	Ch	Regulates gene expression through targeting by miRNA168.	[110]
	SDP	AtSDP	*Arabidopsis*	Ch	Participates in the processing of chloroplast 16S, 23S, 4.5S, and 5S rRNA.	[111,112]

Nc, nucleus; Cy, cytoplasm; Ch, chloroplast; Mt, mitochondria; SGs, stress granules; -, unknown.

## Data Availability

Not applicable.
